# A real-world pharmacovigilance study of FDA adverse event reporting system (FAERS) events for bimekizumab

**DOI:** 10.3389/fphar.2025.1714173

**Published:** 2026-01-05

**Authors:** Zhuomiao Lin, Xihui Yu, Meiqing Yang, Jialan Xu, Jiahong Zhong

**Affiliations:** 1 Department of Clinical Pharmacy, Meizhou People’s Hospital (Huangtang Hospital), Meizhou, China; 2 Department of Pharmacy, The Second Affiliated Hospital of Shantou University Medical College, Shantou, China; 3 Joint Shantou International Eye Center, Shantou University and the Chinese University of Hong Kong, Shantou, China; 4 Department of Pharmacy, First Hospital of Quanzhou Affiliated to Fujian Medical University, Quanzhou, China

**Keywords:** bimekizumab, FAERS, immunomodulator, adverse events, psoriasis

## Abstract

**Background:**

Bimekizumab, a humanized monoclonal antibody, exerts its therapeutic effect by inhibiting interleukin-17A/F and is indicated for the treatment of moderate-to-severe plaque psoriasis in adult patients. However, the long-term safety profile of bimekizumab remains under evaluation. In this study, adverse events were analyzed using data from the US Food and Drug Administration’s Adverse Event Reporting System (FAERS).

**Methods:**

We analyzed adverse event reports in FAERS from the third quarter of 2021 to the fourth quarter of 2024, with bimekizumab identified as the primary suspected drug. The analytical methods included the Reported Odds Ratio, Proportional Reporting Ratio, Bayesian Confidence Propagation Neural Network, and Multi-Item Gamma Poisson Shrinker.

**Results:**

A total of 2,744 suspected adverse event cases with bimekizumab as the major suspected drug were collected from the FAERS database in this study. The results showed that common clinical adverse events of bimekizumab included injection site pain, fatigue, pruritus, headache, arthralgia, rash, pain, oesophageal candidiasis, diarrhea. In addition, we detected probable unexpected adverse events using disproportionality analysis, such as depression.

**Conclusion:**

We identified potential new adverse events associated with bimekizumab through disproportionate analysis of extensive real-world data from the FAERS database. These findings enable healthcare professionals and pharmacists to prioritize effective management of high-risk adverse events, optimize drug utilization in clinical settings, and enhance patient medication safety.

## 1 Introduction

Psoriasis, a chronic non-infectious inflammatory skin disorder, is characterized by well-demarcated scaly erythematous plaques or lesions. These lesions typically involve the trunk, extensor surfaces of limbs, and scalp, with severe cases often progressing to generalized cutaneous involvement (Korman, 2020). Epidemiological evidence indicates that this condition affects approximately 125 million individuals globally (Singh et al., 2021). In recent years, psoriasis has been reclassified as a systemic disease, frequently associated with multi-system comorbidities, including cardiovascular disorders, type 2 diabetes mellitus, inflammatory bowel disease, depressive disorders, and neoplastic diseases (Mrowietz et al., 2024). It has emerged as the second leading cause of skin-related disability worldwide, imposing substantial health burdens and economic costs on both patients and healthcare systems (Koppu et al., 2022). Although the exact pathogenesis of psoriasis remains incompletely understood, accumulating evidence highlights the interleukin-23 (IL-23)/interleukin-17(IL-17) signaling axis as a central driver of disease initiation and progression (Bugaut and Aractingi, 2021). The clinical development of biologic agents targeting these cytokines has significantly improved treatment outcomes and quality of life for psoriasis patients, underscoring the critical role of this pathway in therapeutic intervention (Maronese et al., 2024).

Bimekizumab, a humanized monoclonal immunoglobulin G1 (IgG1) antibody, represents a novel IL-17 A/F inhibitor (Potestio et al., 2025). This agent potently blocks the interaction with the IL-17RA/IL-17RC receptor complex by selectively neutralizing both IL-17A and IL-17F cytokines (Potestio et al., 2025). Based on data from three phase III RCTs registered on ClinicalTrials.gov (NCT03370133, NCT03410992, NCT03412747), bimekizumab was granted approval by the U.S. Food and Drug Administration (FDA) in 2023 for the treatment of adult patients with moderate-to-severe plaque psoriasis (Reich et al., 2021a; Gordon et al., 2021; Warren et al., 2021; Reich et al., 2021b; Koppu et al., 2022). Collectively, data from these three phase III trials demonstrated that bimekizumab provided sustained and effective symptom improvement in plaque psoriasis, achieving durable skin clearance across long-term follow-up periods (Freitas et al., 2021). Although bimekizumab demonstrated comparable safety and tolerability profiles to other biologic agents, four phase III trials revealed a higher incidence of adverse events (AEs) in bimekizumab-treated patients compared to placebo recipients. The most frequently reported AEs included nasopharyngitis, oral candidiasis, upper respiratory tract infection, and joint pain (Reich et al., 2021a; Gordon et al., 2021; Warren et al., 2021; Reich et al., 2021b).

As a novel selective IL-17 inhibitor, bimekizumab has a limited post-marketing history. Current safety profiles for patients with chronic immune-mediated inflammatory diseases primarily derive from clinical trials and post-authorization observational studies, underscoring the need for real-world evidence to validate long-term safety. Real-world studies can effectively overcome the limitations of clinical trials, such as insufficient sample representation due to strict inclusion and exclusion criteria, and limited statistical power due to small sample size. Therefore, this study was conducted by mining the U.S. Food and Drug Administration Adverse Event Reporting System (FAERS) database. The FAERS database, the largest public pharmacovigilance database in the world, can be used to detect drug adverse events (AEs). This study aimed to assess the post-marketing long-term safety of bimekizumab by analyzing data from the FAERS database. We systematically characterized bimekizumab-related AEs and patient characteristics, with the objective of providing critical evidence to guide its rational clinical use and enhance the safety of psoriasis treatment.

## 2 Materials and methods

### 2.1 Data source and collection

This study utilized the data from the FAERS database to collect all AE reports related to bimekizumab in FAERS from Q1 2004 to Q4 2024. The data were classified in accordance with the standards of Edition 27.0 of the Medical Dictionary of Regulatory Activities (MedDRA), especially for the standardized description of ae using Systemic Organ Class (SOC) and Preferred Terms (PT). In this classification, SOC identifies the category of AE, while PT records the specific event name.

**FIGURE 1:**
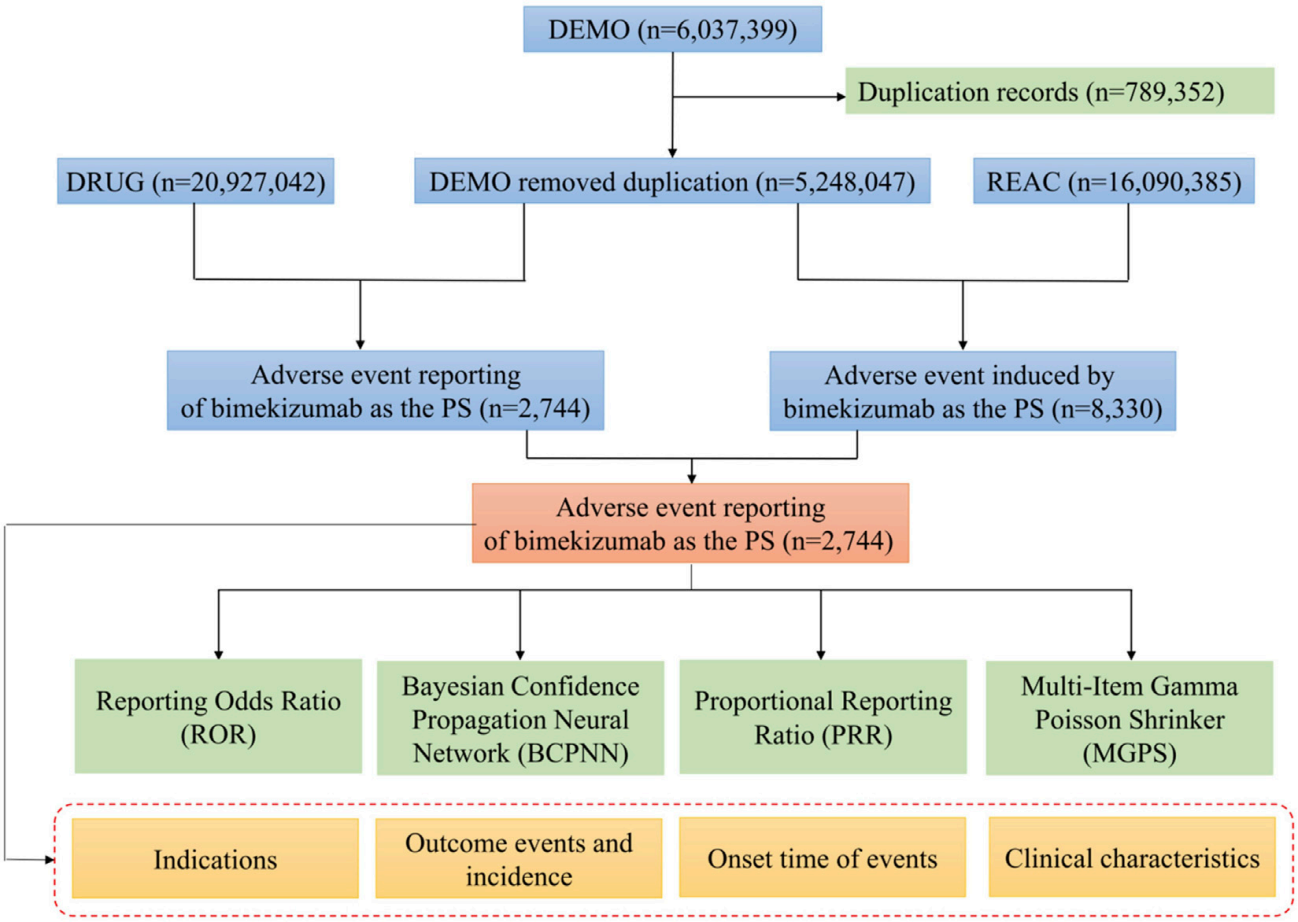
Flow diagram of the study (DEMO, demographic and administrative information; DRUG, drug Information; REAC, preferred terminology for adverse drug reactions; PS, primary suspect drug).

The FAERS data were acquired from the Quarterly Data Extract Files, which are publicly accessible at https://fis.fda.gov/extensions/FPD-QDE-FAERS/FPD-QDE-FAERS.html. We obtain the FAERS data and clean the data via RStudio following the instructions from the FDA. In the process of data extraction, all bimekizumab-related reports from the FAERS database were included. To ensure comprehensive capture of all relevant reports, we filtered bimekizumab by both generic “bimekizumab” and brand name “BIMZELX” in the DRUG table. When referring to the names of AEs in the reports, preferred terms (PTs) from the Medical Dictionary for Regulatory Activities (MedDRA) should be used for consistent encoding. The study included all PTs that fell within the larger category of diseases and infestations known as system-organ classes (SOC). Following data extraction, we implemented a deduplication strategy recommended by the FDA to ensure report uniqueness and accuracy. For records with identical report numbers (CASE ID) but distinct report dates (FDA_DT), only the most recent report was retained. In cases where both the CASE ID and FDA_DT are identical, the entry with the larger main ID was selected to ensure retention of the most complete information. FDA_DT refers to the date when the FDA received the report. CASEID refers to different individuals while PRIMARYID represents the report number. It should be noted that an individual may have multiple reports of AE, so an CASEID could have different PRIMARYID. The larger value of PRIMARYID means that the reported date is more recent. The deduplication process was executed using an R script (version 4.3.3) to efficiently manage large datasets, with all duplicate records imported into Microsoft Excel for manual review to ensure no omissions or errors. A total of 2,744 reports were extracted from the FAERS database (Figure 1).

### 2.2 Statistical analysis

The correlation between bimekizumab and AE was evaluated by statistical algorithms such as reported odds ratio (ROR), proportional reported ratio (PRR), Bayesian belief Propagation neural network (BCPNN), and multi-item Gamma-Poisson contraltor (MGPS). ROR and PRR methods with high sensitivity were selected in order to mine more ignored adverse reaction signals in this paper. At the same time, in order to avoid the misleading of false positive signals, we chose BCPNN and MGPS methods with high specificity to ensure the robustness of the results. A preferred terminology is considered a positive signal if it simultaneously meets the thresholds of all four algorithms, and the equations and criteria for the four algorithms are detailed in Table 1. Our investigation centered on AE signals meeting the specific thresholds established by each algorithm. A signal representing a new adverse event was defined as any significant adverse event not previously listed in the product information. The onset time was defined as the time interval between the occurrence of the AE (EVENT_DT) and the initiation of bimekizumab treatment (START_DT). Reports with incorrect data entries (such as EVENT_DT before START_DT or containing invalid dates) were excluded from the analysis. The onset time was summarized using the median and interquartile range. Statistical analysis and data processing were conducted using R 4.3.3, Navicat Premium 15 and Microsoft Excel 2021.

**TABLE 1:** Calculation formula and standard of signal detection.

Algorithm	Calculation formula	Criterion
ROR	$\text{ROR}=\frac{a/c}{b/d}=\frac{ad}{bc}$	a ≥3
	$95\%\text{CI}=e^{\ln\left(ROR\right)\pm 1.96\sqrt{\frac{1}{a}+\frac{1}{b}+\frac{1}{c}+\frac{1}{d}}}$	95%CI (lower limit) > 1
PRR	$\text{PRR}=\frac{a/\left(a+b\right)}{c/\left(c+d\right)}$	a ≥3, PRR ≥2
	$\chi^{2}=\frac{{\left(ad-bc\right)}^{2}\left(a+b+c+d\right)}{\left(a+b\right)\left(a+c\right)\left(c+d\right)\left(b+d\right)}$	χ^2^ ≥ 4
EBGM	$\text{EGBM}=\frac{a\left(a+b+c+d\right)}{\left(a+b\right)\left(a+c\right)}$	EBGM05 > 2
	$\text{EBGM}05=e^{\ln\left(EBGM\right)-1.64\sqrt{\frac{1}{a}+\frac{1}{b}+\frac{1}{c}+\frac{1}{d}}}$	
BCPNN	$\text{IC}=log_{2}\frac{a\left(a+b+c+d\right)}{\left(a+b\right)\left(a+c\right)}$	a ≥3
	$E\left(IC\right)=log_{2}\frac{\left(a+\gamma_{11}\right)\left(N+\alpha \right)\left(N+\beta \right)}{\left(N+\gamma \right)\left(a+b+\alpha_{1}\right)\left(a+c+\beta_{1}\right)}$ $V\left(IC\right)=\frac{1}{{\left(\ln2\right)}^{2}}\left[\frac{N-a+\gamma -\gamma_{11}}{\left(a+\gamma_{11}\right)\left(N+1+\gamma \right)}+\frac{N-a-b+\alpha -\alpha_{1}}{\left(a+b+\alpha_{1}\right)\left(N+1+\alpha \right)}+\frac{N-a-c+\beta -\beta_{1}}{\left(a+c+\beta_{1}\right)\left(N+1+\beta \right)}\right]$ $\gamma =\gamma_{11}\frac{\left(N+\alpha \right)\left(N+\beta \right)}{\left(a+b+\alpha_{1}\right)\left(a+c+\beta_{1}\right)}$ $95\%CI=E\left(IC\right)\pm 1.96\sqrt{V\left(IC\right)}$ Where α = α_1_+α_2_, β = β_1_+β_2_, N = a+b + c + d, and the value of α_1_, α_2_, β_1_, β_2_ and γ_11_ were defined as 1	The lower limit of 95%CI (IC025) > 0

Abbreviations: a, number of reports containing both the target drug and target adverse drug reaction; b, number of reports containing other adverse drug reaction of the target drug; c, number of reports containing the target adverse drug reaction of other drugs; d, number of reports containing other drugs and other adverse drug reactions. 95%CI, 95% confidence interval; χ2, chi-squared; IC, information component; IC025, the lower limit of 95% CI, of the IC; EBGM05, the lower limit of 90% CI, of the EBGM.

## 3 Results

### 3.1 General characteristics

A total of 2,744 reports related to Bimekizumab were extracted separately from the FAERS database. Demographic characteristics, including gender, weight, reporter occupation, reporting country, and clinical outcomes, were summarized in Table 2. In terms of gender distribution, females comprised 54.3% of cases, while males accounted for 38.1%. Although the majority of reported ages were unknown, the largest age-specific subgroup was 18–65 years (25.4%). Medical professionals submitted the majority of reports (46.4%), with the United States being the most frequent source country (72.4%). Apart from the unclear outcome, the most reported were other serious (important) medical events (35.0%). Notably, the number of bimekizumab-related AE reports demonstrated a significant increase between 2021 and 2024.

**TABLE 2:** Characteristics of reports associated with bimekizumab from the FAERS database (Q1 2004-Q4 2024).

Factors	Number of events (%)
Case reports	2,744
Gender	
Female	1,489 (54.3%)
Male	1,045 (38.1%)
Unknown	210 (7.7%)
Age (year)	
<18	24 (0.9%)
≥18 and <65	697 (25.4%)
≥65 and ≤85	153 (5.6%)
>85	3 (0.1%)
Unknown	1867 (68.0%)
Reporter	
Consumer	925 (33.7%)
Health Professional	1,272 (46.4%)
Physician	514 (18.7%)
Pharmacist	33 (1.2%)
Reporter country	
United States	1988 (72.4%)
Canada	298 (10.9%)
Others	458 (16.7)
Outcome	
Hospitalization (initial or prolonged)	339 (12.4%)
Death	26 (0.9%)
Congenital Anomaly	1 (0.0%)
Life threatening	21 (0.8%)
Disability	11 (0.4%)
Other serious (important medical event)	960 (35.0%)
Required Intervention to Prevent Permanent Impairment/Damage	1 (0.0%)
Unknown	1,385 (50.5%)
Reporting year	
2021	0 (0)
2022	3 (0.1)
2023	68 (2.5)
2024	2,673 (94.4)

### 3.2 Signal detection

#### 3.2.1 Signals of SOC (SOC)

**FIGURE 2:**
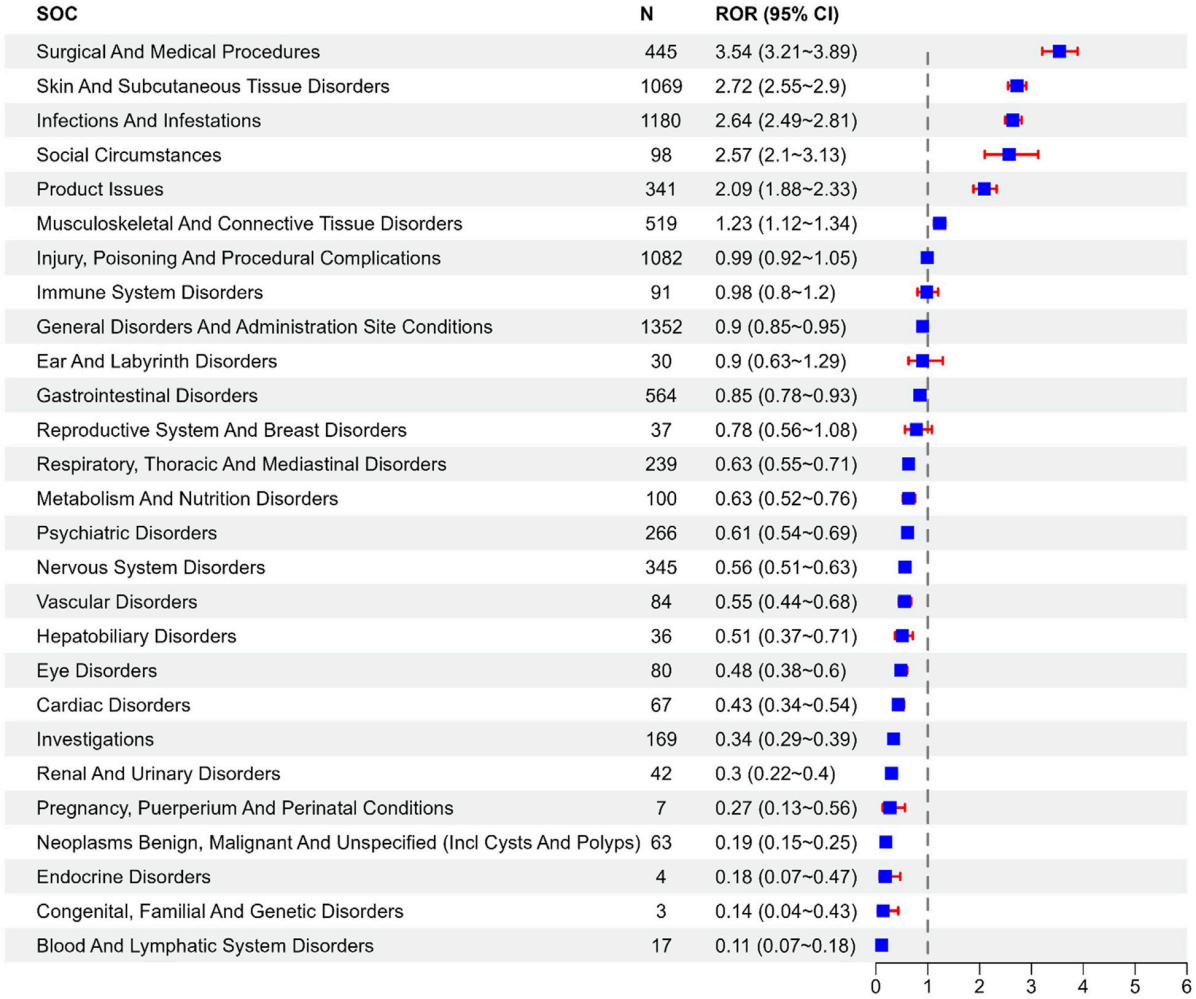
Reporting odds ratios with 95% CI for adverse events at the System Organ Class level.

Bimekizumab-related AEs exhibited different signal intensities in different SOCs, as shown in Figure 2. The analysis revealed that bimekizumab-related AEs affected 27 organ systems in the FAERS database. The top four corresponding SOCs were general disorders and administration site conditions, infections and infestations, injury, poisoning and procedural complications, skin and subcutaneous tissue disorders ranked by the number of AE reports, as shown in Table 3.

**TABLE 3:** The signal strength of bimekizumab at the System Organ Class (SOC) level.

System organ class (SOC)	Case number	ROR (95% CI)	PRR (χ^2^)	EBGM (EBGM05)	IC (IC025)
infections and infestations	1,180	2.64 (2.49–2.81)	2.41 (1,034.55)	2.41 (2.29)	1.27 (1.18)
renal and urinary disorders	42	0.3 (0.22–0.4)	0.3 (69.85)	0.3 (0.23)	−1.74 (-2.18)
injury, poisoning and procedural complications	1,082	0.99 (0.92–1.05)	0.99 (0.21)	0.99 (0.94)	−0.02 (-0.11)
skin and subcutaneous tissue disorders	1,069	2.72 (2.55–2.9)	2.5 (1,014.74)	2.5 (2.37)	1.32 (1.23)
investigations	169	0.34 (0.29–0.39)	0.35 (217.01)	0.35 (0.31)	−1.52 (-1.74)
nervous system disorders	345	0.56 (0.51–0.63)	0.58 (111.36)	0.58 (0.53)	−0.78 (-0.94)
gastrointestinal disorders	564	0.85 (0.78–0.93)	0.86 (13.07)	0.86 (0.8)	−0.21 (-0.34)
surgical and medical procedures	445	3.54 (3.21–3.89)	3.4 (764.64)	3.4 (3.13)	1.76 (1.62)
respiratory, thoracic and mediastinal disorders	239	0.63 (0.55–0.71)	0.64 (51.35)	0.64 (0.57)	−0.65 (-0.84)
general disorders and administration site conditions	1,352	0.9 (0.85–0.95)	0.92 (12.79)	0.92 (0.87)	−0.13 (-0.21)
metabolism and nutrition disorders	100	0.63 (0.52–0.76)	0.63 (21.81)	0.63 (0.54)	−0.66 (-0.95)
musculoskeletal and connective tissue disorders	519	1.23 (1.12–1.34)	1.21 (20.48)	1.21 (1.13)	0.28 (0.15)
neoplasms benign, malignant and unspecified (incl cysts and polyps)	63	0.19 (0.15–0.25)	0.2 (209.74)	0.2 (0.16)	−2.32 (-2.68)
psychiatric disorders	266	0.61 (0.54–0.69)	0.62 (64.32)	0.62 (0.56)	−0.68 (-0.86)
ear and labyrinth disorders	30	0.9 (0.63–1.29)	0.9 (0.34)	0.9 (0.67)	−0.15 (-0.67)
immune system disorders	91	0.98 (0.8–1.2)	0.98 (0.04)	0.98 (0.82)	−0.03 (-0.33)
pregnancy, puerperium and perinatal conditions	7	0.27 (0.13–0.56)	0.27 (13.88)	0.27 (0.15)	−1.89 (-2.91)
blood and lymphatic system disorders	17	0.11 (0.07–0.18)	0.12 (116.8)	0.12 (0.08)	−3.11 (-3.79)
reproductive system and breast disorders	37	0.78 (0.56–1.08)	0.78 (2.31)	0.78 (0.6)	−0.36 (-0.83)
product issues	341	2.09 (1.88–2.33)	2.05 (186.83)	2.05 (1.87)	1.03 (0.88)
cardiac disorders	67	0.43 (0.34–0.54)	0.43 (51.32)	0.43 (0.35)	−1.21 (-1.57)
vascular disorders	84	0.55 (0.44–0.68)	0.56 (30.51)	0.56 (0.46)	−0.85 (-1.16)
hepatobiliary disorders	36	0.51 (0.37–0.71)	0.52 (16.39)	0.52 (0.39)	−0.95 (-1.43)
congenital, familial and genetic disorders	3	0.14 (0.04–0.43)	0.14 (15.98)	0.14 (0.05)	−2.84 (-4.29)
social circumstances	98	2.57 (2.1–3.13)	2.55 (92.59)	2.55 (2.16)	1.35 (1.06)
eye disorders	80	0.48 (0.38–0.6)	0.48 (44.98)	0.48 (0.4)	−1.05 (-1.37)
endocrine disorders	4	0.18 (0.07–0.47)	0.18 (15.49)	0.18 (0.08)	−2.51 (-3.8)

**FIGURE 3:**
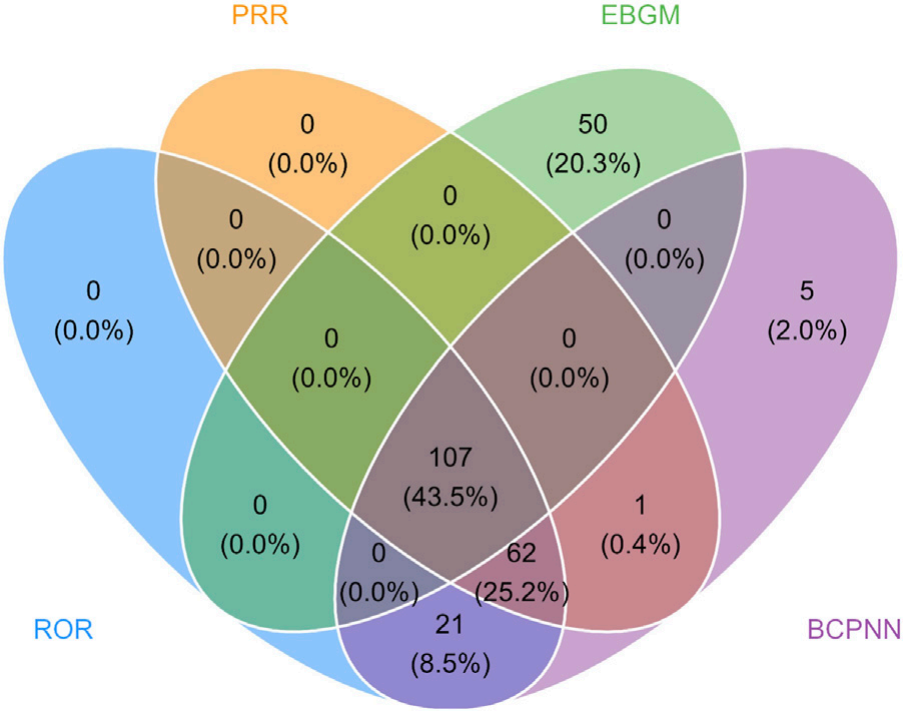
Venn diagram of preferred term (PT) signals meeting the criteria of four algorithms.

In the FAERS database, the four algorithms identified 107 PT signals related to bimekizumab, covering eight SOCs, and the detailed screening process was shown in Figures 3. According to the frequency of occurrence, the top 30 PTs were listed in Table 4, sorted by frequency, and the top 10 most frequently reported PTs were as follows: injection site pain, fatigue, pruritus, headache, arthralgia, rash, pain, oesophageal candidiasis, diarrhea.

**TABLE 4:** Top 30 frequency of adverse events at the PT level for bimekizumab.

SOC	PT (preferred term)	Case number	ROR (95% CI)	PRR (χ^2^)	EBGM (EBGM05)	IC (IC025)
general disorders and administration site conditions	injection site pain	151	3.92 (3.34–4.61)	3.87 (322.5)	3.87 (3.38)	1.95 (1.71)
infections and infestations	oral candidiasis	148	98.15 (83.09–115.93)	96.42 (13313.79)	91.88 (79.93)	6.52 (6.28)
general disorders and administration site conditions	fatigue	110	1 (0.83–1.21)	1 (0)	1 (0.86)	0.01 (-0.27)
skin and subcutaneous tissue disorders	pruritus	99	1.84 (1.51–2.24)	1.83 (37.45)	1.83 (1.55)	0.87 (0.58)
nervous system disorders	headache	98	1.32 (1.09–1.62)	1.32 (7.71)	1.32 (1.12)	0.4 (0.11)
musculoskeletal and connective tissue disorders	arthralgia	80	1.26 (1.01–1.57)	1.26 (4.23)	1.26 (1.05)	0.33 (0.01)
skin and subcutaneous tissue disorders	rash	69	1.19 (0.94–1.51)	1.19 (2.11)	1.19 (0.98)	0.25 (-0.1)
general disorders and administration site conditions	pain	67	0.67 (0.53–0.85)	0.67 (10.88)	0.67 (0.55)	−0.57 (-0.93)
infections and infestations	oesophageal candidiasis	66	150.02 (116.68–192.88)	148.84 (8998.62)	138.26 (112.04)	7.11 (6.74)
gastrointestinal disorders	diarrhoea	65	0.73 (0.57–0.93)	0.73 (6.52)	0.73 (0.6)	−0.45 (-0.81)
infections and infestations	*candida* infection	64	25.26 (19.72–32.36)	25.08 (1,460.86)	24.77 (20.13)	4.63 (4.27)
infections and infestations	pneumonia	60	1.47 (1.14–1.9)	1.47 (8.97)	1.47 (1.19)	0.55 (0.18)
psychiatric disorders	depression	58	2.66 (2.06–3.45)	2.65 (59.77)	2.65 (2.13)	1.41 (1.03)
skin and subcutaneous tissue disorders	acne	46	7.11 (5.32–9.5)	7.07 (239.11)	7.05 (5.53)	2.82 (2.39)
infections and infestations	nasopharyngitis	45	1.66 (1.24–2.23)	1.66 (11.79)	1.66 (1.3)	0.73 (0.3)
gastrointestinal disorders	nausea	44	0.47 (0.35–0.63)	0.47 (25.97)	0.47 (0.37)	−1.08 (-1.51)
general disorders and administration site conditions	illness	42	1.32 (0.97–1.78)	1.31 (3.18)	1.31 (1.02)	0.39 (-0.05)
general disorders and administration site conditions	pyrexia	42	0.95 (0.7–1.28)	0.95 (0.13)	0.95 (0.73)	−0.08 (-0.52)
respiratory, thoracic and mediastinal disorders	cough	39	0.95 (0.69–1.3)	0.95 (0.12)	0.95 (0.73)	−0.08 (-0.54)
infections and infestations	fungal infection	36	8.42 (6.07–11.69)	8.39 (233.37)	8.36 (6.35)	3.06 (2.59)
immune system disorders	hypersensitivity	35	1.54 (1.1–2.15)	1.54 (6.58)	1.54 (1.16)	0.62 (0.14)
infections and infestations	cellulitis	34	5.91 (4.22–8.28)	5.89 (137.64)	5.87 (4.43)	2.55 (2.06)
infections and infestations	infection	33	1.56 (1.11–2.2)	1.56 (6.69)	1.56 (1.17)	0.64 (0.15)
general disorders and administration site conditions	condition aggravated	33	0.64 (0.45–0.89)	0.64 (6.87)	0.64 (0.48)	−0.65 (-1.15)
respiratory, thoracic and mediastinal disorders	oropharyngeal pain	32	2.43 (1.71–3.43)	2.42 (26.68)	2.42 (1.81)	1.27 (0.77)
general disorders and administration site conditions	malaise	32	0.67 (0.47–0.94)	0.67 (5.33)	0.67 (0.5)	−0.58 (-1.09)
infections and infestations	lower respiratory tract infection	32	4.67 (3.3–6.61)	4.66 (91.7)	4.65 (3.47)	2.22 (1.71)
general disorders and administration site conditions	therapeutic response shortened	32	3.96 (2.8–5.61)	3.95 (70.53)	3.95 (2.95)	1.98 (1.48)
infections and infestations	urinary tract infection	31	1.3 (0.92–1.85)	1.3 (2.17)	1.3 (0.97)	0.38 (-0.13)
skin and subcutaneous tissue disorders	hidradenitis	31	16.05 (11.27–22.87)	16 (432.35)	15.87 (11.8)	3.99 (3.48)

#### 3.2.2 Onset time of AEs

**FIGURE 4:**
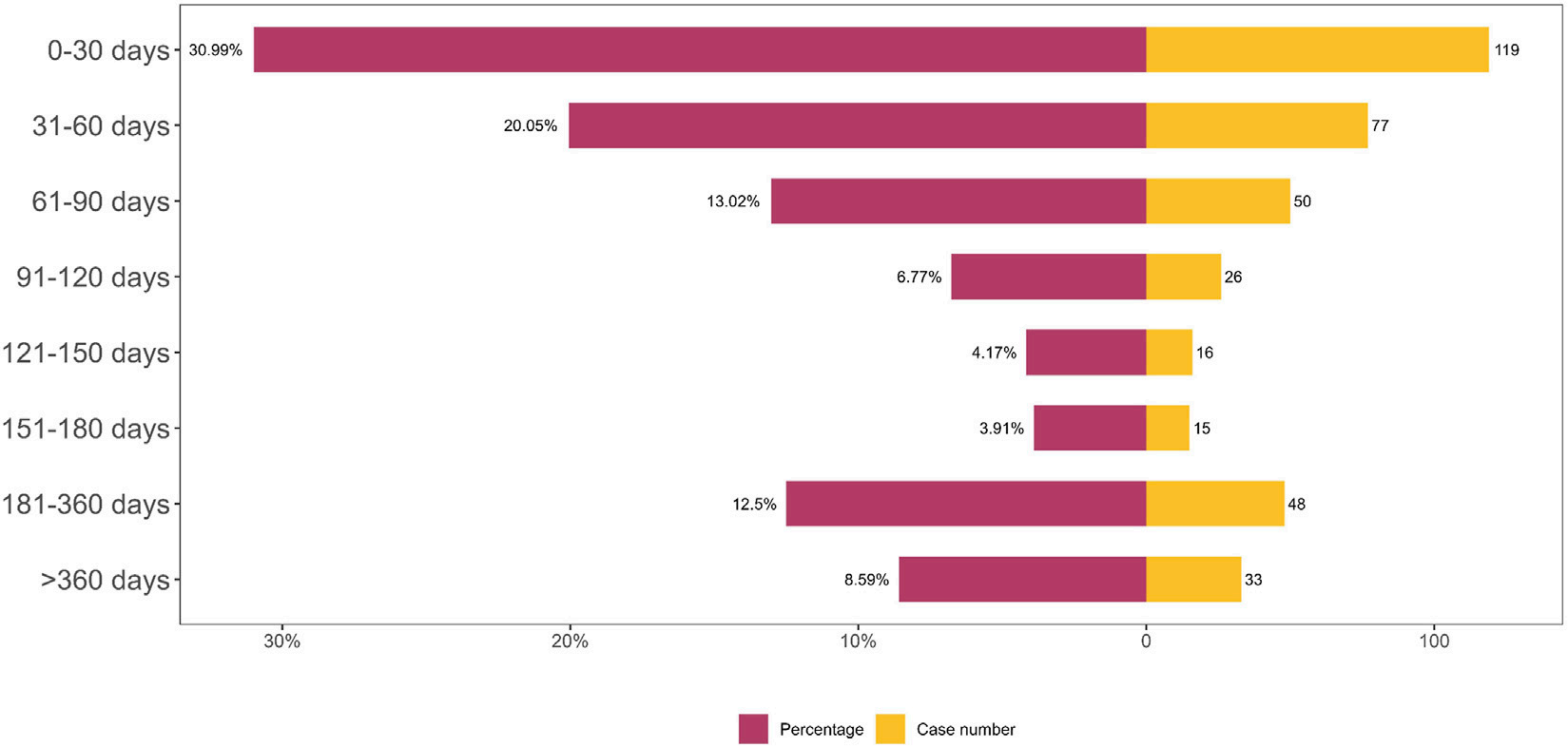
TTO analysis of bimekizumab-related AEs counted in days.

In the FAERS database, a total of 384 records included precise data on AE onset timing. As depicted in Figure 4 the distribution of AE onset times revealed that the majority of cases occurred within the first 30 days of treatment.

## 4 Discussion

Bimekizumab, a novel biologic immunotherapy, exerts its therapeutic effect in plaque psoriasis by selectively neutralizing IL-17A and IL-17F (Koppu et al., 2022). In Phase III clinical trials, bimekizumab exhibited high efficacy and a favorable safety profile in the treatment of psoriasis (Reich et al., 2021a; Gordon et al., 2021; Warren et al., 2021). Furthermore, in a direct comparative study, bimekizumab demonstrated superior efficacy compared to adalimumab, ustekinumab, and secukinumab (Warren et al., 2021; Reich et al., 2021a; Gordon et al., 2021). Notably, the majority of clinical investigations feature short-term follow-up durations, inherently limiting their ability to assess the safety for rare adverse events. Therefore, this real-world pharmacovigilance study represented the most comprehensive and systematic synthesis of global AE reports for bimekizumab in the FAERS database.

In this study, we comprehensively and systematically analyzed AE reports of bimekizumab from the third quarter of 2021 to the fourth quarter of 2024. We found that AE of bimekizumab was more common in women (54.3%) than in men (38.1%). The larger number of reports from female patients may reflect that the incidence of psoriasis in women is slightly higher than that in men (Chiam et al., 2011). Data indicated that the primary age range for bimekizumab-related AEs was 18–65 years, which may correlate with the age distribution of psoriasis onset. Psoriasis is reported to exhibit a bimodal age-of-onset pattern, with 75% of cases occurring in patients younger than 40 years. The mean ages of first onset are approximately 15–20 years and 55–60 years, respectively (Freitas et al., 2021). Furthermore, our study revealed that a substantial proportion of AEs occurred within 60 days and between 181 and 360 days following treatment initiation. These findings underscore the importance of vigilant monitoring during both the early treatment phase and extended follow-up periods. Notably, the United States accounted for the majority of AE reports, suggesting potential disparities in drug safety surveillance across regions and highlighting the need for enhanced awareness and reporting practices in other countries.

Our disproportionality analysis revealed that the most frequently observed AEs of bimekizumab were predominantly clustered in the following SOCs: general disorders and administration site conditions, infections and infestations, injury, poisoning and procedural complications, skin and subcutaneous tissue disorders, which were consistent with the data from the drug instructions and clinical trials (Ruggiero et al., 2022). Among the diseases classified as “infections and infestations” in SOC, the most common ones included: oral candidiasis (n = 148), esophageal candidiasis (n = 66), candidiasis infection (n = 64), and pneumonia (n = 60). In phase III clinical trials, oral candidiasis emerged as the most commonly reported AE in the bimekizumab treatment group. This observation aligned with the drug’s mechanism of action, as bimekizumab selectively inhibits IL-17 A/F, a cytokine pivotal to oral mucosal homeostasis (Freitas et al., 2021). Preclinical and clinical studies have demonstrated that the IL-17/Th17 axis is integral to the oral mucosa’s dual functions of protective immunity and regulated inflammation (Wang et al., 2024). Specifically, this signaling pathway is crucial for maintaining epithelial barrier integrity and orchestrating immune responses against pathogenic infections (Gaffen and Moutsopoulos, 2020). Disrupting IL-17 signaling through bimekizumab therapy may thus compromise host defense mechanisms, contributing to the elevated risk of oral candidiasis (Gaffen and Moutsopoulos, 2020). A phase III clinical trial demonstrated that bimekizumab was associated with a significantly higher incidence of oral and oropharyngeal candidiasis compared with secukinumab (Reich et al., 2021b). Additionally, ixekizumab (another IL-17A inhibitor) was associated with a higher risk of infections than etanercept (TNF-α inhibitor) (Valenzuela et al., 2017). This divergent susceptibility profile further underscores the central role of the IL-17 signaling pathway in mucosal antifungal immunity. The mammalian target of rapamycin (mTOR) signaling pathway, mediated by mTORC1 and mTORC2, regulates critical physiological processes including cell growth, protein synthesis, metabolism, and autophagy. Dysregulation of this pathway is implicated in cancer, metabolic diseases, and autoimmune disorders (Fan et al., 2025; Zhao et al., 2024). As a key upstream regulator, the mTOR pathway is essential for the differentiation of Th17 cells and facilitates IL-17 production via downstream effectors (Michailidou et al., 2025; Nagai et al., 2013). Bimekizumab may exert its therapeutic effects through a synergistic inhibition of IL-17-mediated signaling and disruption of the mTOR activation loop, which is essential for Th17 cell function. This dual blockade likely contributes to an increased risk of impaired host defense. The patients’ susceptibility to infection suggests that we should closely monitor the patient during treatment. Clinicians should implement relevant medical measures if the infection worsens.

Biological agents, including TNF inhibitors, IL-17 inhibitors, and IL-12/23 inhibitors, have demonstrated significant efficacy in the treatment of psoriasis (Ohtsuki et al., 2019). However, with the prolonged clinical use, these agents have drawn growing attention to potential cardiovascular adverse reactions. A meta-analysis including 38 randomized controlled trials indicated no significant evidence that biological agents increase the risk of major adverse cardiovascular events (MACE) during short-term treatment (Rungapiromnan et al., 2017). However, it has been noted that the development process of briakinumab, an inhibitor of IL-12/23, has been hindered by safety issues. Data from briakinumab’s Phase III clinical trials revealed that patients in the treatment arm experienced serious cardiovascular AEs, including myocardial infarction and cardiac death, ultimately leading to the discontinuation of its marketing application process (Gordon et al., 2012). Furthermore, a retrospective analysis of individual case safety reports in European Economic Area and the United Kingdom by the EudraVigilance database shows that heart-related diseases accounted for 3% of suspected drug-related serious adverse events of the IL-23 inhibitor risankizumab over the past 11 years (Ding et al., 2024). These findings underscore the need for ongoing vigilance regarding the long-term cardiovascular safety of biologic agents. Potential signals of cardiovascular events associated with bimekizumab were detected following a comprehensive analysis of real-world post-market data from FAERS. However, the modest severity of these signals and the small number of reported cases suggest that biologic agents have distinct safety profiles. Concurrently, this evidence reinforces the importance of not overlooking the potential for life-threatening adverse reactions, necessitating appropriate therapeutic interventions when clinically indicated.

The drug instructions of bimekizumab also mention that inflammatory bowel disease (IBD) is occasionally observed in patients treated with bimekizumab. IBD has been documented in association with IL-17A inhibitor therapy (Jiang et al., 2023). It is reported that one patient has developed ulcerative colitis after receiving bimekizumab in phase 3 trials (Gordon et al., 2021; Reich et al., 2021a; Griffith et al., 2021). In the comparison of the efficacy of bimekizumab and secukinumab in the treatment of plaque psoriasis, one case of ulcerative colitis was reported in each treatment arm, suggesting that drugs inhibiting IL-17 may induce or worsen IBD (Reich et al., 2021b). Diarrhea is a common clinical manifestation of ulcerative colitis (Kaenkumchorn and Wahbeh, 2020). In our study, AEs classified as gastrointestinal disorders in the SOC included diarrhea and nausea. Previous studies have demonstrated that psoriasis patients exhibit an elevated risk of developing IBD, which may be attributed to the protective role of IL-17A in intestinal homeostasis (Fieldhouse et al., 2020). Inhibition of IL-17 signaling is thought to upregulate proinflammatory cytokines, such as TNF-α, IFN-γ, IL-6, and disrupt intestinal tight junction proteins, thereby compromising epithelial barrier integrity (Deng et al., 2023). The estimated prevalence of IBD in individuals with psoriasis ranges from 1% to 2%, compared to 0.4% in the general population (Eppinga et al., 2017). Therefore, some psoriasis patients treated with IL-17 inhibitors, such as bimekizumab, may develop subclinical IBD as a consequence of therapy. This highlights the importance of vigilance for IBD in patients who experience diarrhea during treatment, and colonoscopy should be considered when clinically indicated. Additionally, prior to bimekizumab treatment, clinicians should conduct a comprehensive medical history evaluation, including a detailed assessment of the patient’s personal and family history of IBD, to aid in the selection of appropriate management plans.

Depression, a new AE classified as psychiatric disorders at the SOC level, has been observed in this study. People with psoriasis often suffer from anxiety or depression (Zafiriou et al., 2021) Emerging evidence indicates that IL-17-producing cell subsets, such as Th17 cells, play a pivotal role in the pathogenesis of depression (Raison et al., 2013). A recent 2-year pharmacovigilance report on a IL-17 inhibitor brodalumab use in the U.S. analyzed data from 2,677 patients, with 25 cases of depression reported (Lebwohl et al., 2021). Safety data from 21 clinical trials demonstrated the occurrence of depressive symptoms in patients treated with ixekizumab (Genovese et al., 2020). Of note, suicidal behavior or self-harm was reported in a subset of these patients (Genovese et al., 2020). Consistent with the study’s findings, depressive symptoms were observed following bimekizumab treatment. Currently, some investigations have explored how bimekizumab might be involved in the pathogenesis of depression. It is hypothesized that its mechanism may relate to blocking the binding of IL-17A subtypes, thereby inducing dysregulation of the inflammatory cytokine microenvironment, damaging the blood-brain barrier, and activating glial cells, etc .,(Mosca et al., 2021; Zafiriou et al., 2021; Sukoff Rizzo et al., 2012). Moreover, these findings suggest that regardless of whether depressive symptoms stem from the disease or treatment-related AEs, close monitoring of patients’ mental status during clinical therapy is essential. Enhancing psychological counseling and intervention carries significant clinical imperative.

Bimekizumab exhibits a favorable therapeutic profile for moderate-to-severe plaque psoriasis, characterized by a rapid onset of action, high levels of skin clearance, and sustained efficacy over the long term. Clinical studies demonstrated that after 10–16 weeks of treatment, bimekizumab was associated with superior efficacy compared to other biological agents, including higher psoriasis area and severity index (PASI) 100 response rates and a larger area under the curve. Additionally, bimekizumab therapy exhibited a more rapid onset of action than most comparator drugs, while maintaining long-term efficacy comparable to the active control (Warren et al., 2024; Armstrong et al., 2022). The management of psoriasis affecting highly visible or functional areas (e.g., scalp, nails, palms, soles) remains a challenge due to its association with severe symptomology and impaired quality of life (Mrowietz and Augustin, 2022; Augustin et al., 2019). Evidence indicated that bimekizumab translated into durable, high-efficacy outcomes in these regions for over 2 years, addressing a key unmet need for long-term disease control (Merola et al., 2024). Bimekizumab demonstrated a manageable safety profile in clinical studies, with upper respiratory tract infections and oral candidiasis being the most frequently reported adverse events (Warren et al., 2024). No new serious safety concerns or increased risk of malignancies were identified (Warren et al., 2024). Consequently, for patients requiring rapid and sustained clearance, especially in difficult-to-treat areas, bimekizumab represents a preferred therapeutic strategy (Merola et al., 2024). Given that our findings suggested a potential association between bimekizumab and depression, alternative biologics like ixekizumab may be a more suitable consideration for patients with pre-existing mental health concerns (Zhou et al., 2024; Schwartzman et al., 2024). In summary, bimekizumab’s dual-targeting mechanism confers distinct clinical advantages. And its safety profile is comparable to other biologic agents, solidifying its position as a leading therapeutic choice in the current management of psoriasis.

This study has some limitations. Firstly, the FAERS database is a global spontaneous reporting system. It suffers from some inherent selection bias, including but not limited to ethnic and geographic variations in reported cases, differences in drug approval timelines and market penetration across regions, public awareness of specific adverse effects, and incomplete capture of all serious adverse event reports. Second, detailed age data were missing in 68% of the AE reports we collected, and 46.4% were from health professionals. Therefore, the potential bias caused by the data should be carefully considered when interpreting the results. Second, the lack of detailed clinical information about the patients, such as comorbidities, severity of the underlying disease, and concomitant medications, further hinders the control of confounding variables. Despite these limitations, the use of the FAERS database for pharmacovigilance studies is inherent. The comprehensive characterization of AE associated with bimekizumab in this study may provide insightful evidence for safe use and further clinical studies.

## 5 Conclusion

In conclusion, this study systematically evaluated the adverse reactions related to bimekizumab through an in-depth analysis of the FAERS database from the third quarter of 2021 to the fourth quarter of 2024. This investigation not only corroborated previously documented safety signals but also uncovered novel potential risks associated with its application in real-world clinical settings. These findings provide a more targeted and comprehensive data base for the clinical practice and public health decision-making of managing bimekizumab. It is worth noting that infection and infections occur frequently and the signal strength is high, indicating the need for further attention and research. The study also revealed new adverse reactions not mentioned in the drug instructions, such as depression, nausea, diarrhoea. These findings suggest that medical professionals should be more cautious when using bimekizumab for treatment, especially being aware of these potential adverse reactions. Appropriate therapeutic interventions should be promptly instituted when AEs occur.

## Data Availability

The original contributions presented in the study are included in the article/supplementary material, further inquiries can be directed to the corresponding authors.

## Abbreviations

FDA, U.S. Food and Drug Administration; FAERS, U.S. Food and Drug Administration’s Adverse Event Reporting System; IL-23, interleukin-23; IL-17, interleukin-17; IgG1, immunoglobulin G1; AEs, adverse events; AEs, drug adverse events; MedDRA, Medical Dictionary of Regulatory Activities; SOC, Systemic Organ Classification; PT, Preferred Terms; DEMO, demographic and administrative information; REAC, preferred terminology for adverse drug reactions; PS, primary suspect drug; ROR, reported odds ratio; PRR, proportional reported ratio; BCPNN, Bayesian belief Propagation neural network; MGPS, multi-item Gamma-Poisson contraltor; MACE, major adverse cardiovascular events; IBD, inflammatory bowel disease; mTOR, mammalian target of rapamycin; PASI, psoriasis area and severity index.
